# Self-confidence, Overconfidence and Prenatal Testosterone Exposure: Evidence from the Lab

**DOI:** 10.3389/fnbeh.2018.00005

**Published:** 2018-01-30

**Authors:** Patricio S. Dalton, Sayantan Ghosal

**Affiliations:** ^1^Economics, Tilburg University, Tilburg, Netherlands; ^2^University of Glasgow, Glasgow, United Kingdom

**Keywords:** 2D:4D, testosterone, neuroeconomics, expectations, overconfidence, self-confidence, goals, C91, D03, D87

## Abstract

This paper examines whether foetal testosterone exposure predicts the extent of confidence and over-confidence in own absolute ability in adulthood. To study this question, we elicited incentive-compatible measures of confidence and over-confidence in the lab and correlate them with measures of right hand 2D:4D, used as as a marker for the strength of prenatal testosterone exposure. We provide evidence that men with higher prenatal testosterone exposure (i.e., low 2D:4D ratio) are less likely to set unrealistically high expectations about their own performance. This in turn helps them to gain higher monetary rewards. Men exposed to low prenatal testosterone levels, instead, set unrealistically high expectations which results in self-defeating behavior.

## 1. Introduction

Self-confidence and overconfidence play a crucial role in people's decisions and welfare. While positive thinking can enhance motivation and improve performance, being overly confident—i.e., believing one is better than one actually is—can be self-defeating (Benabou and Tirole, 2002). Indeed, overconfidence bias has been used to explain phenomena such as business failures (Camerer and Lovallo, 1999), stock market bubbles and excessively frequent trading (Barber and Odean, 2001; Grinblatt and Keloharju, 2009). An important question that arises is what determines the level of self-confidence and overconfidence. It is known that nurture does play a role. Mastering own experiences and observing successful experiences of similar others can influence people's confidence (Bandura, 1977). Does nature play any role too?

We address this question by examining whether prenatal testosterone exposure determines people's confidence and overconfidence about their own ability to perform a rather unfamiliar and challenging task^1^. As a marker for the strength of prenatal testosterone exposure we used the ratio of the length of the index finger to the length of the ring finger (2D:4D) of the right hand. We followed the vast literature started by Manning et al. (1998) which shows that individuals with conditions associated with very high prenatal testosterone levels exhibit significantly smaller 2D:4D (Brown et al., 2002)^2^. To measure confidence and overconfidence, we implemented an incentive-compatible scheme. We introduced participants to an unfamiliar task, and we asked them to report the number of tasks they expected to solve during the experiment. Their total final earnings depended on the precision of their estimate, so subjects had incentives to truthfully report their expected performance (i.e., their confidence in their own ability)^3^. Our experimental design also allowed us to measure subjects' degree of overestimation of their actual performance (i.e., overconfidence) in an incentive-compatible way. We paid the subjects piece-wise during their performance task, so, when performing, they had enough monetary incentives to perform up to their maximal potential. The difference between these two incentive-compatible measures (i.e., expected minus actual performance) constituted our incentive-compatible measure of overconfidence.

We found that, ceteris paribus, male subjects exposed to low prenatal testosterone levels were more likely to overestimate their actual performance. Such overestimation, rather than being a rational strategy to increase motivation and hence performance, showed to be self-defeating. Overconfident participants gained significantly less earnings than participants who were rather conservative in their expectations. This is in line with Benabou and Tirole's (2002) seminal model which predicts that overconfidence can harm welfare but individuals may nevertheless display it. Our paper provides empirical evidence for this theoretical finding and it also suggests a biological origin for such systematic overconfidence.

This paper contributes to three different strands of literatures. First, it contributes to the literature of psychology. Overconfidence is “perhaps the most robust finding in the psychology of judgment” (De Bondt and Thaler, 1995, p. 389). Here we provide evidence that it is—at least partially—biologically determined.

Second, it contributes to the literature of behavioral finance. Inasmuch our experimental results can be extrapolated to the world outside the laboratory, they suggest a plausible link between two well-known empirical finding in finance, namely that overconfident traders earn lower returns than more conservative traders Barber and Odean (2001) and that male traders with lower 2D:4D earn higher long term returns and remain longer time on business (Coates et al., 2009). Our findings would suggest that the higher success of traders with lower 2D:4D might be due to less overconfidence bias. Of course, this is just a conjecture that could be directly tested in the future.

Third, the paper contributes to an emerging literature in economics which studies the relationship between 2D:4D and economic preferences, skills and economic behavior. 2D:4D has been shown to be correlated with social preferences (van den Bergh and Dewitte, 2006; Millet and Dewitte, 2009; Buser, 2012; Brañas-Garza et al., 2013; Galizzi and Nieboer, 2015), risk preferences (Brañas-Garza et al., in press), cooperation in prisoner's dilemma (Sanchez-Pages and Turiegano, 2010), contributions to public goods (Cecchi and Duchoslav, 2016), cognitive reflection (Bosch-Domènech et al., 2014), social integration (Kovárík et al., 2017) and effort provision (Friedl et al., 2018). In the domain of finance, low digit ratio individuals achieve higher trading profits (Coates and Herbert, 2008; Coates et al., 2009), are more likely to self-select into the financial services profession (Sapienza et al., 2009), and are more active and risk-taking traders (Cronqvist et al., 2016)^4^. However, to the best of our knowledge, there is not much work investigating the link between 2D:4D, confidence and overconfidence. Neyse et al. (2016) study the relation between 2D:4D and participants prediction accuracy of their performance in a cognitive reflection test. They found that when using incentivized predictions, males with low digit ratios, on average, are less overconfident about their performance.

The rest of the paper is organized as follows. Section 2 introduces the experimental method. Section 3 describes the data and section 4 introduces the results. Section 5 concludes.

## 2. Methods

We designed an experiment to measure the three variables of interest: (ex-ante) self-confidence, ex-post overconfidence and the second to fourth digit ratio (2D:4D). Through emails and leaflets, we recruited 255 undergraduate and graduate students from the University of Warwick. We conducted twelve sessions with approximately twenty students each. Each session lasted 60 min. The average payment was £ 14 including a show up fee of £ 5. In each session, the sequence of the experiment was as follows. Once each subject read and signed the consent form, the experimenter would read out loud the experimental instructions, which included a description of the task and the monetary payments^5^. Participants were informed that they had 20 min to complete the same task and that they would be paid 100 points (equivalent to £ 1) per completed task. Subjects were given 1 min of practice time to get familiar with the task and after that, we elicited their self-confidence in the following way^6^. We asked them to predict the number of tasks they expected to successfully complete in the 20 min of performance time. The answer to that question constituted our measure of self-confidence. In section 2.1 below we describe the incentive-compatible mechanism of self-confidence elicitation. Once the subjects reported their prediction, they started performing the task for 20 min. When they finished, they were asked to fill in a questionnaire, they were paid and their right hands were scanned. Below we describe in more detail the manner in which self-confidence, overconfidence and the 2D:4D were measured.

### 2.1. Confidence, overconfidence, and incentives scheme

Self-confidence is broadly defined as a feeling of trust in one's ability, quality and judgment. The literature of social psychology has operationalized this broad concept using two related constructs: “perceived self-efficacy”and “outcome expectations.” Perceived self-efficacy is a judgment of capability to execute given types of performances; outcome expectations are judgments about the anticipated outcomes that would arise from such performances (Bandura, 1977, 1986)^7^.

Both psychological concepts are usually measured with surveys compounded of several rather broad statements to which the respondents have to agree or disagree following a Likert scale. For example, perceived self-efficacy scales include items such as “I can solve most problems if I invest the necessary effort”or “I can usually handle whatever comes my way.” Outcome expectancy scales contain statements of the type “If I quit smoking I will save money”or “If I quit smoking I will gain weight.”

Although these scales have been proven to be useful in many settings, they were not appropriate for the purpose of this paper for the following reasons. First, we required a unidimensional and easily interpretable measure of how confident the person was about his/her capacity to perform an unfamiliar task in the lab. These scales are rather multidimensional and general. Second, this paper also aimed at measuring overconfidence, so we needed to be able to evaluate how far were expectations from actual performance. The existing psychological scales are simply not developed to measure this construct. Finally, we needed to capture the true expectations of own performance and at the same time, we wanted to ensure that subjects performed up to their maximum capacity during performance time. To achieve this, we provided subjects with the following monetary incentive scheme. Subjects were asked to solve a practice task for 1 min. Once the practice period was over, their self-confidence *C* was measured by asking them to report how many tasks they expected to solve during the 20-min period. The subject received a piece rate of 100 points per solved task, *P*, minus 40 points for each task that he mispredicted when estimating future performance:

100×P−40×|C−P|

The misprediction penalty provided the subjects with an incentive to truthfully report their perceived performance distribution. Note that this scheme implies that the effective piece rate of performance was 140 points for each successfully completed task as long as they stay below their estimate and 60 points for each successfully completed task thereafter. Hence, truthful elicitation of self-confidence implied that the marginal incentive during the performance period decrease (though remain positive) once reaching the estimated number of tasks. For this reason, we chose a generous exchange rate from points to money (£ 0.01 per point) to ensure that even 60 points represented a salient reward and the subject had high enough incentives to continue putting effort. Moreover, once the subject reached his estimate, it meant that he/she figured out the way to solve the task, so the marginal cost of effort put thereafter is close to zero. Note that even if the participants chose to stop before the 20 min, they would have had to wait doing nothing until the 20 min have passed. Hence, they had two options once they reached *C*: to stop and wait doing nothing, or continue implementing mechanically the algorithm that they had already figured out and earn money. Almost all students chose the second option, so by revealed preferences, the marginal benefit of solving the task was higher than the marginal cost. As already argued, once the task has been figured out, the marginal cost of an additional task is close to zero^8^.

Above and beyond confidence, we were interested in measuring the degree of overconfidence. Moore and Healy (2008) defines overconfidence as the overestimation of one's actual performance and we apply this definition for this paper^9^. Like self-confidence, the degree of overconfidence is usually measured with answers to survey questionnaires, in a non-incentivised way. For the same reasons exposed above, we used an incentive compatible measure of overconfidence. A person was considered to be overconfident when he/she expected to perform better than his/her actual performance. This measure pins down overconfidence in an incentive compatible way because subjects had monetary incentives to both, announce their expectations as accurately as possible and perform as good as possible.

### 2.2. 2D:4D and other measures

At the end of the experiment, we scanned the right hand of each subject, we measured the length of their second and fourth finger, and calculated their ratio (2D:4D ratio)^10^. Finger length was measured by two independent research assistants using a digital caliper. All data analysis was done using the average of the two independent measures of ratios^11^.

In addition to the variables of interest, we collected independent data in a post-experiment questionnaire to construct variables that were used as controls in our regressions. In particular, we elicit risk attitudes using the Eckel and Grossman (2002) method in a non-incentivized way. This method involves a single choice among six hypothetical gambles. The gambles differ in expected return and variance. Each gamble has two possible outcomes with fifty percent probabilities of each occurring. The higher the gamble, the higher expected payoff but also the higher the risk involved^12^.

We also used the General Self-Efficacy Scale (Schwarzer and Jerusalem, 1995) to measure generalized perceived self-efficacy (see Appendix D in Supplementary Material). This Likert-type scale consists of 10 statements. Subjects were asked to indicate how true they think each statement was for them. The scale was validated in several studies and widely used internationally (Schwarzer and Born, 1997). It captures, in a general way, the belief that one can perform well in a novel or difficult tasks.

### 2.3. The task

For our experiment, we chose a computerized puzzle which consisted of a modified version of the so-called “Tower of Hanoi”(ToH) puzzle. The standard ToH consists of three straight bars, and a number of disks of different sizes which can slide onto any bar^13^. The puzzle starts with the disks in a pile in ascending order of size on one bar, the biggest at the bottom, thus making a conical shape. The challenge of the puzzle is to move the entire pile of disks to another bar, respecting the following rules: (a) only one disk can be moved at a time, (b) each move consists of taking the upper disk from one of the bars and sliding it onto another bar, on top of the other disks that may already be present on that bar and (c) no disk may be placed on top of a smaller disk. We used a slightly modified version of the original ToH to increase difficulty. In our case, instead of having disks of different sizes, there were disks of different colors. The rule was to always preserve the original order of colors of the disks (pink, green, blue, turquoise, brown). For example, brown could be moved on top of any other disks, but green could only be moved on top of the pink, etc^14^.

We chose this puzzle for several reasons. First, the rules of the task were easy to understand, which reduced the possibility of noise. Second, the task had a unique solution (involving thirty one moves), computed by backward induction. Third, it was quite unfamiliar to subjects and it constituted an Eureka-type of problem (Cooper and Kagel, 2005): it appeared to be challenging at first glance, but simple to solve once the algorithm is figured out. This is a desirable property for a self-confidence and overconfidence measure, since it allowed us to elicit expectations within a setting in which people had imperfect knowledge of their own abilities^15^. Indeed, in our experiment, only five subjects managed to solve the task in the practice time, but all eventually made it during the performance time.

## 3. Data

Two hundred and fifty five students from Warwick University participated in the study. The sample was proportionally balanced by gender^16^. Five subjects who solved the task in the practice time were excluded from all the analysis. We decided to exclude them because their prediction of expected performance would not involve any level of uncertainty about their capacity to perform. Further, we excluded one outlier with an overconfidence level forty times higher than the mean and two subjects who did not report their gender. Therefore, the final sample we analyze consisted of two hundred and forty nine subjects.

**Figure 1:**
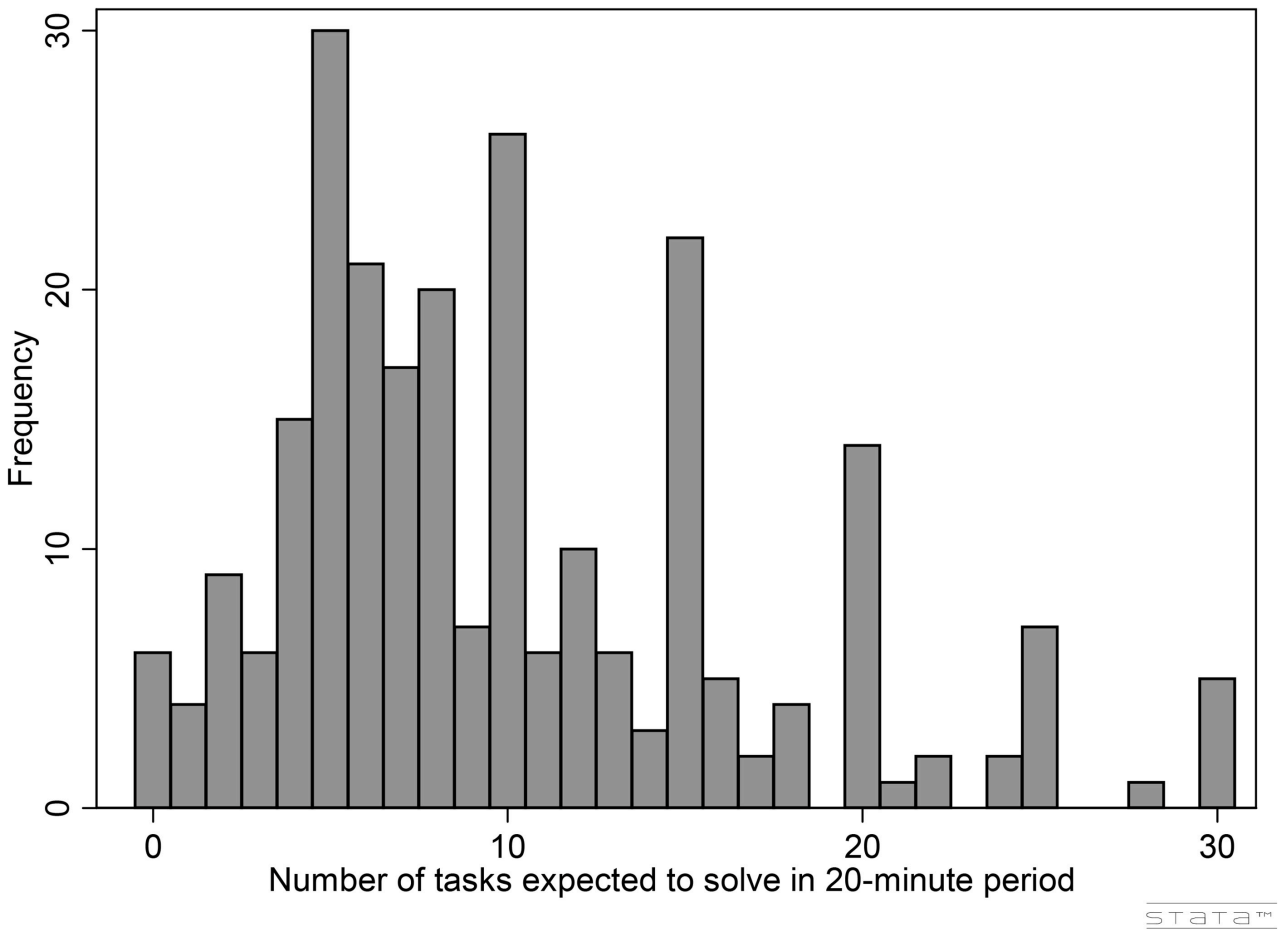
Self-confidence measure: frequency.

Table 1 shows the summary statistics of our experimental measure of self-confidence. On average, subjects expected to solve about ten ToHs in 20 min, with a standard deviation of about six. As Figure 1 shows, the frequency distribution of confidence in our data is quite disperse and rather skewed to the right, with a median at eight, a mode at five, a minimum at zero and a maximum at thirty. Finally, although this paper is not about gender differences, it is worth noticing that in average men expected to perform 40% better than women (*P* <0.01)^17^.

**Table 1:** Self-confidence: summary statistics.

	**Mean**	**Std. Dev**.	**Min**	**Max**	**Obs**
Whole sample	10.14343	6.629279	0	30	249
Female	8.48062	5.976648	0	30	129
Male	12.01667	6.848889	0	30	120

We also looked at other variables that we expected to be positively correlated with our measure of self-confidence (see Table 2). As expected, we observed a significant positive correlation with Schwarzer and Jerusalem (1995) general measure of perceived self-efficacy (*P* <0.01)^18^. Likewise, self-confidence was positively correlated with some proxies of the ability to solve the task such as being enrolled in a mathematical oriented degree (*P* <0.01) and being familiar with the task (*P* <0.10). We also looked at its correlation with our risk attitude index, since one could expect that risk averse subjects set lower expectations. However we don't find evidence of a link between these two variables.

**Table 2:** Self-confidence: Pair-wise correlations.

**Construct**	**Variable**	**Self-confidence**
	Maths oriented degree	0.1759^***^
Ability	Familiarity with the task	0.119^*^
Beliefs	Self-efficacy	0.1635^***^
Preferences	Risk-Attitude	0.0039

*** *significant at 1%*,

* *significant at 10%*.

**Figure 2:**
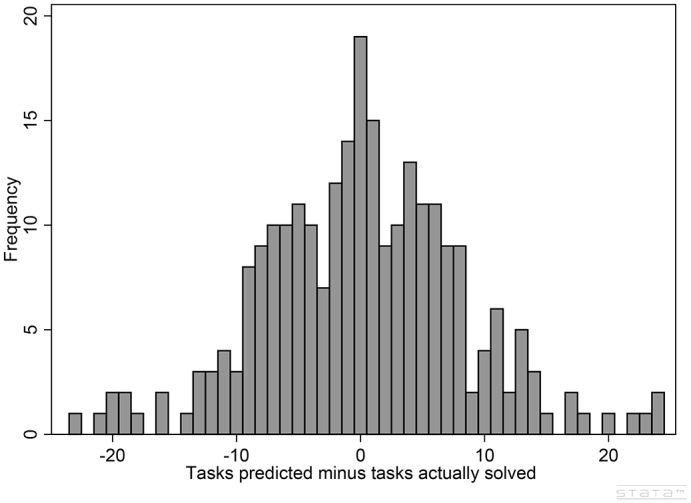
Prediction minus actual performance.

Table 3 and Figure 2 describe the data on overconfidence. Recall that those subjects whose expectations were higher (respectively lower) than their actual performance are classified as overconfident (respectively underconfident). As it can be seen in Table 3, the sample is equally divided between these two groups of subjects, with only 7% of the subjects performing exactly the way they expected to perform. Interestingly, the number of overconfident (hence underconfident) subjects is equal for men and women.

**Table 3:** Predicted and actual performance.

**Type**	**Predicted vs. actual performance**	**Total**	**Female**	**Male**
Underconfident	Predicted < Actual Performance	114	58	56
Precise	Predicted = Actual Performance	19	7	12
Overconfident	Predicted > Actual Performance	116	59	57
		249	124	125

Finally, Table 4 summarizes the data on 2D:4D ratio. The average of 0.96 as well as the gender differences are in accordance with standard findings in the literature: male ratios are typically shorter than those of female.

**Table 4:** 2D/4D: Summary statistics.

	**Mean**	**Std. Dev**.	**Min**	**Max**	**Obs**
Whole sample	0.960252	0.03248	0.8467053	1.041442	249
Female^***^	0.968466	0.028316	0.8968218	1.041442	128
Male	0.951187	0.034542	0.8467053	1.028392	119

## 4. Results

### 4.1. Self-confidence and prenatal testosterone exposure

In Table 5 we report the results of a linear regression analysis examining the relation between our measure of self-confidence and the digit ratio^19^. Self-confidence was significatively positively correlated with the digit ratio, suggesting that high self-confidence was associated with low prenatal testosterone exposure. When data were analyzed separately for men and women, we found that the effect was entirely driven by men. Also, as expected, men exhibited significantly higher self-confidence than women (*P* <0.01).

**Table 5:** OLS regressions of 2D:4D on self-confidence.

	**Both genders**				**Women**				**Men**			
	**I**	**II**	**III**	**IV**	**I**	**II**	**III**	**IV**	**I**	**II**	**III**	**IV**
Average digit ratio	26.749^**^	30.298^**^	30.513^**^	29.594^**^	12.32	13.01	13.07	13.10	36.734^**^	39.409^*^	39.201^*^	38.099^*^
	(11.11)	(11.68)	(11.06)	(10.80)	(16.45)	(15.40)	(15.60)	(15.69)	(16.71)	(18.43)	(18.40)	(17.86)
Gender: Male = 1	3.931^***^	3.540^***^	3.572^***^	3.310^***^	–	–	–	–	–	–	–	–
	(0.96)	(0.96)	(0.94)	(0.88)	–	–	–	–	–	–	–	–
Familiarity with task		0.994	1.006	0.918		1.74	1.737	1.511		−0.19	−0.17	−0.12
		(1.19)	(1.20)	(1.19)		(2.11)	(2.19)	(2.18)		(1.06)	(0.94)	(0.95)
Math degree		1.676^*^	1.573	1.612		−0.332	−0.335	−0.282		3.231^**^	3.134^**^	3.166^**^
		(0.93)	(1.04)	(1.07)		(1.95)	(1.92)	(1.97)		(1.19)	(1.24)	(1.22)
Risk attitude index			−0.16	−0.19			−0.005	−0.059			−0.38	−0.37
			(0.28)	(0.29)			(0.37)	(0.39)			(0.43)	(0.42)
Self-efficacy				0.180^*^				0.160^*^				0.12
				(0.09)				(0.089)				(0.24)
Observations	247	245	244	244	128	128	128	128	119	117	116	116

*This table shows OLS regressions of number of repetitions of tasks expected to solve in 20 min after 1 min of practice time on the 2D:4D digit ratio. All regressions include sessions fixed effects and robust standard errors clustered by session are reported in brackets*.

*** *significant at 1%*,

** significant at 5%,

* *significant at 10%*.

The correlation between prenatal testosterone exposure and self-confidence may not reflect a causal relation between these variables but rather be due to a third variable, independently correlated with testosterone and self-confidence. For example, it may be that subjects enrolled in a mathematics oriented degree or who are familiar with the ToH, are also those who have been exposed to lower prenatal testosterone (i.e., high 2D:4D) and because of their better knowledge (and not directly because of the prenatal testosterone exposure) they expected to perform better than those with a low 2D:4D. However, when we control for these two factors, the estimated coefficient of self-confidence on 2D:4D remains substantially the same (Table 5, column II). The same happens with the risk attitude index and self-efficacy. When we include these variables in the regression, the association between prenatal testosterone exposure and self-confidence remains virtually unchanged (Table 5, columns III and IV). Interestingly, the degree of previous expertise with the task (measured with proxies such as being enrolled in a maths degree or familiarity with the task), has a significant positive correlation with male (rather than female) self-confidence, whereas perceived self-efficacy is significatively positively correlated with female (rather than male) self-confidence.

### 4.2. Overconfidence and prenatal testosterone exposure

Table 6 reports results on the relation between our measure of overconfidence and digit ratio. Recall that overconfidence is defined as expectations minus actual performance, so this variable takes positive values when the person is overconfident, and is increasing in the degree of confidence. When we regressed this measure on digit ratio, we found that they were significatively positive correlated, suggesting that high overconfidence was associated with low prenatal testosterone exposure (Table 6). After controlling for possible confounding variables, like previous experience with the task, risk attitude index and self-efficacy, the association between prenatal testosterone exposure and overconfidence became even stronger. (Table 6, columns III and IV). Again, we found this effect only in men. Also, as expected, we found that the higher the degree of previous expertise with the task and the higher the self-efficacy, the lower the overconfidence^20^.

**Table 6:** OLS regression of 2D:4D on expectations minus actual performance.

	**Both genders**				**Women**				**Men**			
	**I**	**II**	**III**	**IV**	**I**	**II**	**III**	**IV**	**I**	**II**	**III**	**IV**
Average digit ratio	34.179^**^	33.265^**^	30.645^**^	31.545^**^	10.938	7.49	1.257	0.613	41.952^**^	48.214^***^	48.417^***^	50.334^***^
	(14.50)	(13.05)	(13.18)	(14.14)	(23.33)	(23.32)	(23.15)	(22.93)	(17.91)	(13.61)	(14.96)	(16.62)
Gender: Male = 1	0.585	1.2	1.659^*^	1.946^*^								
	(1.37)	(1.07)	(0.93)	(0.95)								
Familiarity with task		−5.897^***^	−5.537^***^	−5.417^***^		−3.888	−3.901	−3.6		−7.054^***^	−6.525^***^	−6.575^***^
		(1.38)	(1.42)	(1.43)		(2.48)	(2.43)	(2.49)		(0.94)	(1.04)	(1.05)
Math degree			−2.655^**^	−2.746^**^			−2.432	−2.471			−2.250^*^	−2.416^*^
			(1.06)	(0.99)			(2.16)	(2.10)			(1.22)	(1.32)
Risk attitude index				0.02				0.121				0.126
				(0.35)				(0.44)				(0.50)
Self−efficacy				−0.210^**^				−0.195				−0.238
				(0.08)				(0.14)				(0.16)
Observations	247	245	244	244	128	128	128	128	119	117	117	116

*This table shows OLS Regressions of a measure of expectations—actual performance on the 2D:4D digit ratio. All regressions include sessions fixed effects and robust standard errors are reported in brackets*.

*** *significant at 1%*,

** *significant at 5%*,

* *significant at 10%*.

**Table 7:** Ordered logit regression of 2D:4D on under/over-confidence.

	**Both genders**				**Women**				**Men**			
	**I**	**II**	**III**	**IV**	**I**	**II**	**III**	**IV**	**I**	**II**	**III**	**IV**
Average digit ratio	7.019^**^	7.237^*^	7.115^*^	6.913^*^	3.797	3.227	2.544	2.388	8.440^*^	10.039^**^	10.042^**^	11.221^**^
	(3.58)	(3.95)	(4.00)	(4.19)	(6.68)	(6.92)	(6.97)	(7.18)	(4.80)	(4.42)	(4.40)	(4.98)
Gender: Male = 1	0.193	0.317^*^	0.336^*^	0.327^*^	.	.	.	.	.	.	.	.
	(0.22)	(0.19)	(0.18)	(0.17)	.	.	.	.	.	.	.	.
Familiarity with task		−1.280^***^	−1.265^***^	−1.264^***^		−1.093^*^	−1.099^*^	−1.081^*^		−1.404^***^	−1.401^***^	−1.478^***^
		(0.31)	(0.31)	(0.31)		(0.59)	(0.59)	(0.58)		(0.40)	(0.41)	(0.42)
Math degree			−0.122	−0.133			−0.268	−0.26			−0.012	−0.07
			(0.30)	(0.30)			(0.40)	(0.42)			(0.43)	(0.45)
Risk attitude index				0.009				0.022				−0.061
				(0.08)				(0.09)				(0.13)
Self−efficacy				−0.038				−0.013				−0.098^*^
				(0.03)				(0.05)				(0.06)
Observations	247	245	244	244	128	128	128	128	119	117	117	116

*This table shows Ordered Logit Regressions of a variable that takes value 0 if Predicted < Actual Performance, 1 if Predicted = Actual Performance and 2 if Predicted >Actual Performance on the 2D:4D digit ratio. All regressions include sessions fixed effects and robust standard errors clustered by session are reported in brackets*.

*** *significant at 1%*,

** *significant at 5%*,

* *significant at 10%*.

### 4.3. Overconfidence and experimental earnings

So far we have shown that men who were exposed to higher prenatal testosterone in their mothers' womb were less likely to be overconfident. An important question that still remains unanswered regards the welfare effects of overconfidence. Was being overconfident good or bad for the subjects? Did overconfident subjects earn more money in the experiment than non-overconfident subjects?

As pointed out by Benabou and Tirole (2002), the answer is not straightforward. On the one hand, setting high expectations can improve earnings by motivating higher effort and hence improving performance. On the other hand, setting excessively high expectations can only increase the cost of not reaching them. Thus, whether overconfidence is in the end a good or a bad strategy is an empirical question. We examined this question by regressing an overconfidence dummy on the final experimental earnings (see Table 8). Our regressions confirm that being overconfident was on average a bad strategy in our experiment. Non-overconfident subjects who set their expectations below their actual potential ended up winning on average eight to nine British pounds more than overconfident subjects^21^. These results are true for both, men and women, and controlling for a series of possible confounders. The magnitude of the cost of overconfidence on earnings was very high: it more than doubled the cost of not having previous experience with the task. Interestingly, the 2D:4D ratio did not affect earnings directly, but trough its effect on self-confidence.

**Table 8:** OLS regression of under/over-confidence on actual earnings.

	**Both Genders**		**Women**		**Men**	
	**I**	**II**	**I**	**II**	**I**	**II**
Exceeded Expectations	8.750^***^	7.573^***^	7.774^***^	7.229^***^	8.851^***^	7.312^***^
	(0.76)	(0.73)	(1.01)	(0.93)	(1.21)	(1.26)
Correct Expectations	4.461^***^	4.512^***^	3.869^***^	3.249^***^	5.549^***^	7.453^***^
	(0.52)	(0.76)	(1.26)	(0.92)	(1.56)	(1.86)
Gender: Male = 1	3.282^***^	1.865^**^	–	–	–	–
	(1.08)	(0.91)	–	–	–	–
Familiarity with task		3.209^**^		2.416^*^		3.550^***^
		1.06		(1.35)		(1.26)
Math degree		3.560^***^		1.227		5.026^***^
		(0.98)		(1.70)		1.10
Risk attitude index		−0.2		−0.053		−0.577^*^
		(0.22)		(0.21)		(0.33)
Self-efficacy		0.285^***^		0.242^**^		0.261
		(0.06)		(0.09)		(0.18)
Average digit ratio		10.32		13.36		4.91
		(11.21)		(15.95)		(14.67)
Observations	247	244	128	128	119	116

*Exceeded expectations is a dummy variable that takes value 1 if Expectations < Actual Performance and zero otherwise. Correct expectations is a dummy variable that takes value 1 if Expectations = Actual Performance and zero otherwise. The benchmark variable for comparison is unreached expectations or overconfidence (i.e., if Expectations >Actual Performance). The dependent variable is final experimental earnings measured in GBP. All regressions include sessions fixed effects and robust standard errors clustered by session are reported in brackets*.

*** *significant at 1%*,

** *significant at 5%*,

* *significant at 10%*.

The subjects who performed better in the lab seemed to have pursued a strategy that the psychologists know as “defensive pessimism”: setting low expectations in uncertain situations to harness anxiety and thus perform better. This strategy was also discussed in the economic model of Benabou and Tirole (2002). In their theory, “defensive pessimism” comes as a result from assuming that ability is a substitute rather than a complement of effort in generating future pay-offs. This gives the person an incentive to discount or repress signals of high ability, as these would increase the temptation to “coast” or “slack off.” In other words, considering the possibility of failure may motivate higher effort to avoid that possibility, and it is a rational strategy to follow inasmuch it increases performance. This is, indeed, what we observe in our experimental data: overconfident subjects gained substantially lower earnings than subjects who set more modestly their expectations. Overconfidence was self-defeating.

## 5. Conclusion

This paper examines the biological determinants of self-confidence and overconfidence. We provide evidence that men with higher prenatal testosterone exposure (i.e., low 2D:4D ratio) are less likely to set unrealistically high expectations about their own performance. Importantly, we also show that such bias has normative implications: overconfidence was detrimental for individuals' earnings. Our results are in line with the findings in Neyse et al. (2016) when they use incentive compatible measures of confidence and over-confidence. Both pieces of independent evidence using different tasks and samples confer further validation to our findings that men with low 2D:4D ratio are less overconfident.

The evidence in this paper can be understood as a plausible explanation of why male financial traders with higher prenatal testosterone exposure remain longer on business or have higher long term profits (Coates et al., 2009). According to our findings, these traders may be less likely to suffer from overconfidence bias, and this helps them to be more successful in the long run. This interpretation is consistent with the empirical findings of Barber and Odean (2001), who show that overconfidence is negatively correlated with traders financial returns^22^.

Our paper also provides an alternative plausible channel through which prenatal testosterone exposure may affect behavior and outcomes in other settings. For instance, prenatal testosterone has been shown to be positively correlated with performance in a range of sports. The main explanation put forward is that it promotes the development of male fighting and competitiveness, which are useful traits to succeed in sports (Manning and Taylor, 2001). The evidence presented here suggests another alternative explanation: men with high prenatal testosterone exposure may succeed in sports because they may use “defensive pessimism”strategies. That is, they may set low expectations to harness anxiety and hence perform better.

## Ethics statement

This study was carried out in accordance with the recommendations of “the Ethics committee in the Faculty of Social Studies at the University of Warwick” with written informed consent from all subjects. All subjects gave written informed consent in accordance with the Declaration of Helsinki. The protocol was approved by the “Ethics committee in the Faculty of Social Studies at the University of Warwick.”

## Author contributions

All authors listed, have made substantial, direct and intellectual contribution to the work, and approved it for publication.

### Conflict of interest statement

The authors declare that the research was conducted in the absence of any commercial or financial relationships that could be construed as a potential conflict of interest.

## Footnotes

^1^Prenatal testosterone exposure has been shown to have important organizing effects on brain development, several psychological traits and behavior (see Tobet and Baum, 1987).

^2^The most direct evidence for the link between 2D:4D and prenatal testosterone exposure comes from Lutchmaya et al. (2004) who measure foetal oestrogen and testosterone levels before birth and record digit lengths at age two. They find that the right-hand digit ratio is significantly correlated with prenatal testosterone levels and the ratio of testosterone to oestrogen levels. See also Zheng and Cohn (2011).

^3^The incentive-compatible scheme of payments we used was also implemented by Mobius and Rosenblat's (2006) to measure self-confidence in a lab setting. Next section describes in detail the mechanism.

^4^Outside of economics, 2D:4D has been found to be correlated with many traits including reproductive success (Manning et al., 2000), sexual orientation (Robinson and Manning, 2000) and competitiveness in sports (Manning and Taylor, 2001).

^5^See Appendix A in Supplementary Material for the instructions and appendices B and C in Supplementary Material for a snapshot of the screen the subjects saw.

^6^1 min was only enough to understand what the task was about, but was not enough to understand how to fully solve it, except for someone who had previous expertise with a similar task. Out of the 257 subjects, only 5 subjects managed to solve the task during the practice time and we excluded them from our analysis. We explain this in more detail in section 3.

^7^Perceived self-efficacy is a very different concept to self-esteem. While perceived self-efficacy is a judgment of capability, self-esteem is a judgment of worth (Bandura, 1977, p. 309).

^8^We are not the first using this elicitation scheme to measure a decision-maker's incentive-compatible absolute self-confidence and performance. We use exactly the same incentive scheme proposed Mobius and Rosenblat's (2006) influential paper. There is other literature eliciting measures of relative self-confidence, that is, estimates of how much individuals expect to be above of below some sample statistics (e.g., median). However, in this paper, we are interested in absolute rather than relative self-confidence.

^9^Overconfidence has also been defined in the literature as the overplacement of one's performance relative to others and as the overestimation of the precision in one's knowledge (Moore and Healy, 2008).

^10^2D:4D was determined from right-hand measurements only, because right-hand digit ratios have been shown previously to display more robust sex differences and are thus thought to be more sensitive to prenatal androgens.

^11^Both independent measures displayed a high repeatability (intraclass correlation 0.875). The results if we used the two measurements separately are qualitatively the same.

^12^Since we did not provide material incentives to elicit risk preferences we label our proxy measure as risk attitude index.

^13^The standard ToH has been extensively studied by cognitive psychologists but very rarely used in economics (McDaniel and Rutström, 2001).

^14^A screenshot of the computerized puzzle can be seen in Appendix C (Supplementary Material).

^15^Imperfect knowledge of own ability is one of the key assumptions made by Benabou and Tirole (2002) to model self-confidence.

^16^32% of men and 21% of women reported to have played a similar game before, while 39% of men and 18% of women were enrolled in a maths related subject.

^17^This and all the tests reported hereafter are two sided.

^18^This correlation should be taken with caution though, since the measure of self-efficacy could be contaminated by the experience of each subject in the experiment.

^19^Given that self-confidence is a count variable, we replicated our analysis using Negative Binomial Regressions and our results do not change. We chose Negative Binomial instead of Poisson regressions due to over dispersion in our data (variance greater than mean).

^20^In addition, we ran an Ordered Logit regression where the dependent variable took value zero if the predicted performance was lower than the actual performance, one if it was equal and two if it was higher. As shown in Table 7, the results remain qualitatively the same.

^21^Note that given that we created the dummies *Exceeded and Correct Expectations*, the benchmark variable for comparisons is *Unreached Expectations*.

^22^The other alternative explanations to Coates, Gurnell and Rustichini (2009) findings rely on risk preferences or preferences for competition. However, the evidence on the link between these two preferences and 2D:4D is mixed. While Sapienza et al. (2009), Apicella et al. (2008), and Schipper (2015) find no significant correlations between risk preferences and the digit ratio, Brañas-Garza et al. (in press) find that subjects with lower digit ratios tend to choose riskier lotteries. Furthermore, Pearson and Schipper (2012) finds no correlation between 2D:4D and competitive behavior in markets.
