# Supplementary material for: Self-confidence, Overconfidence and Prenatal Testosterone Exposure: Evidence from the Lab
Source: Front Behav Neurosci. 2018 Jan 30;12:5. doi: 10.3389/fnbeh.2018.00005 (PMC5797613; doi:10.3389/fnbeh.2018.00005)
Supplement: Supplementary file 1 [file DataSheet1.pdf]

Appendix

A Session Instructions

[As Subjects (Ss) arrive, the experimenter welcomes them, hands them an ID card and invites them to sit in the computer desk corresponding to the ID. Tell them to wait and not to login in the computer. Once everyone is sat, the experimenter asks the students to read the information sheet and consent forms and sign up if they agree. The experimenter reads the following:]

Pre-instructions

Welcome to this research on individual decision making. My name is xxx. These are my assistants xxx and xxx who are going to help me in this research project today. As you were informed in the recruitment process, you will be asked to perform multiple rounds of a task and fill in a questionnaire. Your total estimated participation time for today is 60 minutes. Before we begin, please read the information sheet and consent form that are placed on your desk, and put your signature on the consent form if you agree to participate. Once you are done, raise your hand and one of our assistants will come to your desk to collect the form.

[The Consent form notes that if they stay in the room, they are agreeing to participate. If Ss refse to participate, then pay the show-up fee and send them on their way. Once all the consent forms have been collected the experimenter reads the following instruction at loud.]

Thank you for agreeing to participate.

[the experimenter activates the login page]

Now you will see the login page on your screen. Please use the computer ID card you have received from us to login. Once you are logged in, you will see a set of instructions. Once you finish reading the instructions, you will have to click on the tab "play the game" at the top of the page. From now on you will advance the session through your own input on the screen and you will not receive any further oral instruction from me. So please follow the instructions on the screen very carefully. If you have any questions at any point please raise your hands and one of us will come to you to assist you privately.

Instructions

Today's session is part of a research project at the University of Warwick, Economics Department. You will receive £ 5 as a participation fee as well as additional earnings depending on your individual performance during this session. Your total earnings will be paid to you privately in cash at the end of this session. From now on, you are requested not to communicate with the other participants. If you have any questions, please raise your hand and one of us will answer your questions privately. Please do not ask them out aloud.

You will be asked to perform multiple repetitions of a task that will appear on your computer screen.

In the first part of this session, you will have the opportunity to practice this task in order to get familiar with it. You will be given 1 minute of practice time.

After your practice time is over, we will ask you to complete this task as many times as you can in 20 minutes. Before you do so, we will ask you to predict, as accurately as you can, the number of times you think you can successfully complete the task in 20 minutes.

Your final compensation will be determined by your participation fee £ 5 plus additional earnings based on your total points, where every 100 points are worth £ 1.

The total points you earn will be equal to your performance points minus a prediction error penalty, where:

Performance points: 100 points for each time that you successfully complete the task in the 20 minutes allotted.

Prediction error penalty: A penalty of 40 points each for each completion above or below your prediction. For instance, if your actual performance exceeds – or falls short – of your predicted number of completions by say 5 times, your prediction penalty will be 200 (or 5 x 40) points in either case. Therefore, the best way to maximize your earnings is to predict your performance as accurately as you can.

Clearly, your prediction may not be exactly right. If, while performing the task, you reach your predicted number and still have time left during the 20 minutes allocated, remember that each additional completion will still earn you a net of 60 additional points (100 performance points for the additional completion - 40 penalty points for being above your prediction).

You must fully complete the task each time before you can start a new one. Our central computer will record your total number of completions in the time allowed and will use this number, together with your initial prediction, to compute the total amount due to you. This amount will be paid in full at the end of the session.

If you have any doubts please raise your hand now. One of us will come to you and answer your question privately. Please don't ask at loud.

[After all concerns have been addressed privately, the experimenter continues reading the instructions:]

After the assigned 20 minutes are over, you will be requested to complete a questionnaire. Once you have completed it, please wait until you are called upon to collect your final payment at the front desk.

Now you can start with the session. Please click "Play the Game" tab at the top of this page and follow the instructions on the screen.

B Puzzle Instructions Screen

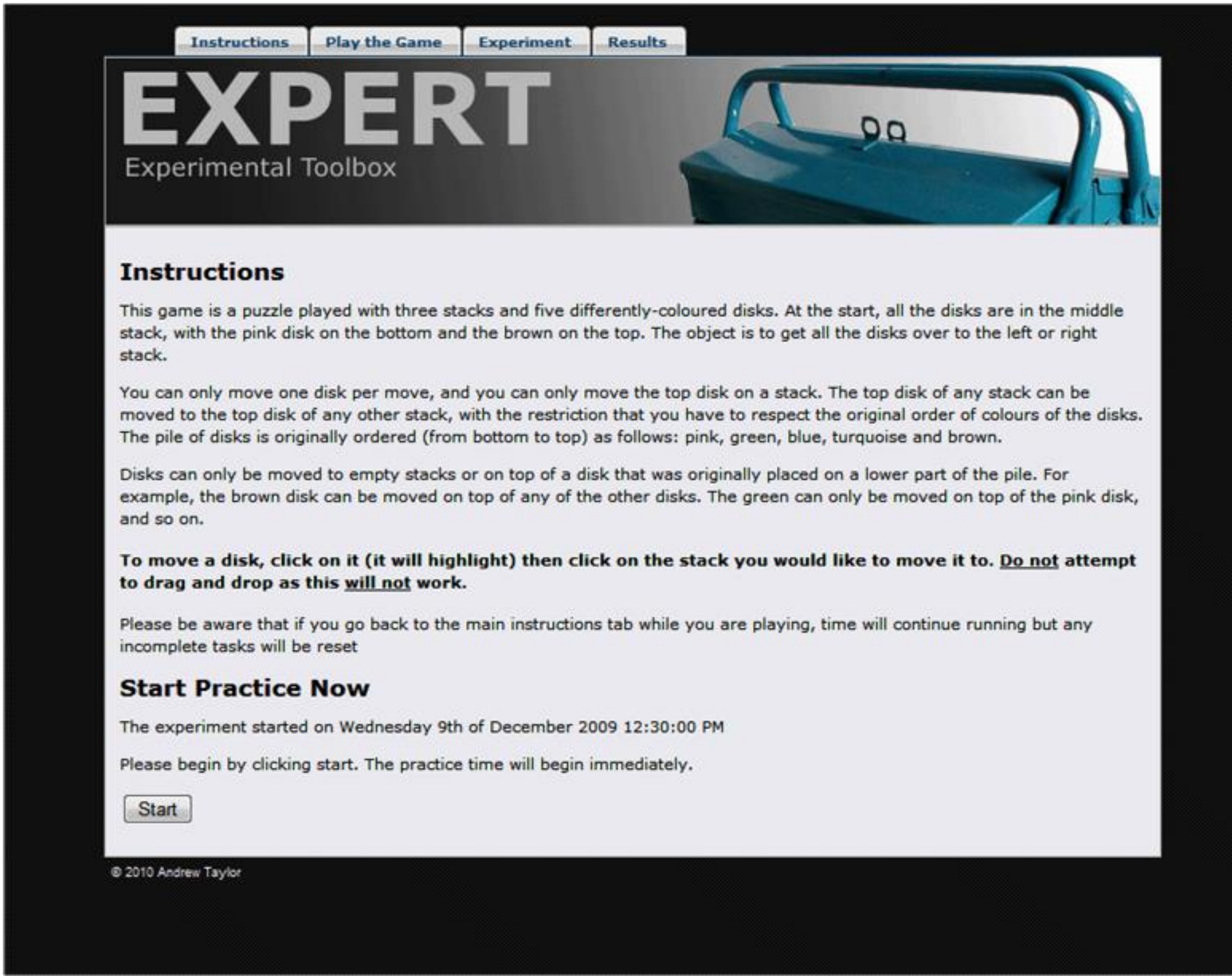

C Puzzle Practice Screen

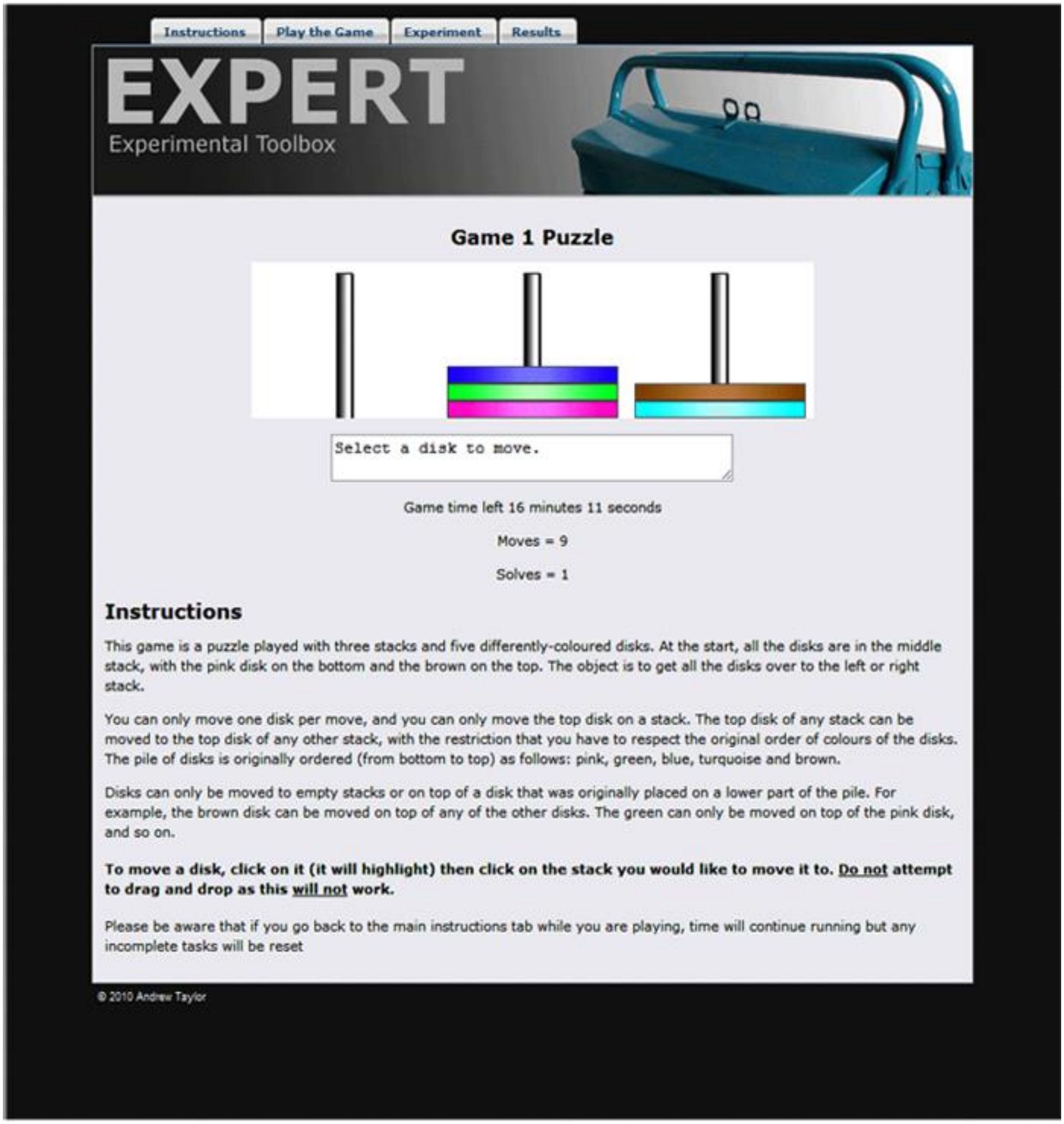

D Self-Efficacy Scale (Schwarzer and Jerusalem, 1995)

For each of the following ten statements indicate how true you think each statement is for you. (1 = not at all true, 2 = hardly true, 3 = moderately true, 4 = exactly true) (write your answer in the space left at the beginning of each statement)

1) .... I can always manage to solve difficult problems if I try hard enough.

2) .... If someone opposes me, I can find the means and ways to get what I want.

3) .... It is easy for me to stick to my aims and accomplish my goals.

4) .... I am confident that I could deal efficiently with unexpected events.

5) .... Thanks to my resourcefulness, I know how to handle unforeseen situations.

6) .... I can solve most problems if I invest the necessary effort.

7) .... I can remain calm when facing difficulties because I can rely on my coping abilities.

8) .... When I am confronted with a problem, I can usually find several solutions.

9) .... If I am in trouble, I can usually think of a solution.

10)........ I can usually handle whatever comes my way.

Scoring: Responses are made on a 4-point scale. Sum up the responses to all 10 items to yield the final composite score with a range from 10 to 40.
